# Dynamic Expression of Palmitoylation Regulators across Human Organ Development and Cancers Based on Bioinformatics

**DOI:** 10.3390/cimb44100306

**Published:** 2022-09-27

**Authors:** Zixian Jia, Deyu Long, Yingcui Yu

**Affiliations:** 1 College of Life Sciences, Northwest A&F University, Xianyang 712100, China; 2 College of Natural Resources and Environment, Northwest A&F University, Xianyang 712100, China

**Keywords:** palmitoylation regulators, organ development, cancer, prognosis

## Abstract

Protein palmitoylation is a reversible modification process that links palmitate to cysteine residues via a reversible thioester bond. Palmitoylation exerts an important role in human organ development and tumor progression. However, a comprehensive landscape regarding the dynamic expression of palmitoylation regulators in human organ development remains unclear. In this study, we analyzed the dynamic expression of palmitoylation regulators in seven organ development and eight cancer types based on bioinformatics. We found that the expression levels of most palmitoylation regulators were altered after birth. In particular, *ZDHHC7/20/21* exhibited converse expression patterns in multiple cancer types. Survival analysis showed that the poor prognosis in patients with kidney renal clear carcinoma (KIRC) is related to low expression of *ZDHHC7/20/21*, and a high expression of *ZDHHC7/20/21* is related to worse survival in patients with liver hepatocellular carcinoma (LIHC). Furthermore, we found that the expression of *ZDHHC7* is associated with infiltration levels of some types of immune cells in the tumor microenvironment (TME), and we explored the relationship between *ZDHHC7* expression and immune checkpoint (ICP) genes across 33 cancer types. In addition, gene set enrichment analysis (GSEA) results indicated that *ZDHHC7* might regulate different genes to mediate the same pathway in different organs. In summary, the comprehensive analysis of palmitoylation regulators reveals their functions in human organ development and cancer, which may provide new insights for developing new tumor markers.

## 1. Introduction

Palmitoylation is an essential and reversible post-translational modification that plays crucial roles in a variety of biological processes, including the regulation of protein stability, membrane trafficking, and enzymatic activity [1,2,3]. The dynamic palmitoylation modification is performed by domain families that apply the modification (writers), remove the modification (erasers) and bind to the modified sites (readers). The “writers” mainly include the aspartate–histidine–histidine–cysteine (DHHC) family (*ZDHHC1-24*, except for *ZDHHC10*) [4]. The “erasers” mainly include the acyl protein thioesterases (APTs), protein palmitoyl thioesterases (PPTs) [4,5] and ABHD17 family (*ABHD17A*, *ABHD17B*, *ABHD17C*) [6].

Numerous studies have shown that palmitoylation regulators participate in organ development and are dysregulated in cancers. For example, *ZDHHC16* (*Aph2*) plays a vital role in regulating heart function and heart development [7]. Similarly, studies have shown that *PPT1* deficiency is one of the causes of neuronal ceroid lipofuscinosis (NCL), suggesting a unique function for *PPT1* in brain development [8]. Decreased *ZDHHC2* expression was observed in LIHC and related to the metastasis and recurrence of LIHC [9]. The *ZDHHC5* was overexpressed in p53-mutated glioma and contributed to the progression of p53-mutated glioma [10]. Chen et al. have identified *ZDHHC9* to be upregulated in colon adenocarcinoma (COAD) and serves as a target for anticancer drug design [11]. A recent article has shown that poor prognosis in patients with glioma is related to high co-expression levels of *ZDHHC18* and *ZDHHC23* [12]. However, there has been no systematic understanding that investigates the roles of palmitoylation regulators during human organ development and multiple cancer types.

This study comprehensively analyzed palmitoylation regulators across seven human organs’ development and eight cancer types. The results showed that expression levels of some palmitoylation regulators significantly changed after birth and also in cancer patients. In addition, we systematically explored the association of *ZDHHC7* expression levels with the TME and found that *ZDHHC7* might be a potential prognostic and immunological pan-cancer biomarker. Finally, we evaluated the potential biological pathways affected by *ZDHHC7* across human organ development and cancers. This study might provide new insights into their roles in human organ development and cancer.

## 2. Materials and Methods

### 2.1. Collection of Palmitoylation Regulators

The gene list of palmitoylation regulators was collected from previously published articles [4,6], including *ZDHHC1-24* (except *ZDHHC10*), *PPT1*, *PPT2*, *APT1*, *APT2*, *APTL1*, *ABHD17A*, *ABHD17B*, and *ABHD17C*. GeneCards database enables researchers to inquire and inter-connect a broad range of human genes, diseases, variants, proteins, cells and biological pathways [13]. Based on the rich annotation of human genes within the GeneCards database (https://www.genecards.org/, accessed on 20 September 2021), these palmitoylation regulators’ gene symbols were converted into Ensemble gene IDs and HGNC symbols.

### 2.2. Gene Expression across Seven Human Organ Development

The transcriptome data across human organ development were obtained from previously published literature and downloaded from ArrayExpress (accession code E-MTAB-6814), involving 297 human samples [14]. The data included seven human organs: brain, cerebellum, heart, kidney, testis, ovary, and liver. The data consisted of prenatal samples from 4 weeks post-conception (WPC) to 20 WPC and postnatal samples from neonates to seniors. Gene expression levels were calculated as fragments per kilobase of exon model per million mapped reads.

### 2.3. Cancer Datasets

We selected eight cancer types for analysis based on the seven organs in human development, including KIRC, kidney renal papillary cell carcinoma (KIRP), kidney chromophobe (KICH), brain lower grade glioma (LGG), glioblastoma multiforme (GBM), ovarian serous cystadenocarcinoma (OV), LIHC, and testicular germ cell tumors (TGCT). UCSC Xena (https://xenabrowser.net/, accessed on 2 October 2021) is a genomics database which includes multi-omics data and phenotype data and provides visual analysis for many public cancer datasets [15]. The RNA-seq data of eight cancer types were downloaded from the UCSC Xena [16,17,18].

### 2.4. Differential Analysis

The Wilcoxon rank sum test was performed to evaluate the differences in expression levels of palmitoylation regulators in tumor and normal samples. The Benjamini and Hochberg method corrected the *p* value [19]. The criteria for selecting differential genes were log2 |Fold-change| ≥ 1 and *p*-adjust < 0.01. The results were visualized using the ggplot2 (version 3.3.0) package in R software. The *p* < 0.05 was considered significant.

### 2.5. Genome Alteration of Palmitoylation Regulators

cBio Cancer Genomics Portal (c-BioPortal, http://cbioportal.org, accessed on 15 October 2021) is an interactive website that explores, visualizes, and analyzes multidimensional cancer genomic data [20]. In this study, the genome alterations of palmitoylation regulators were also identified by the cBioPortal online platform. We utilized the “Cancer Types Summary” module to analyze the alternation frequency of palmitoylation regulators and the results are shown in a barplot. Next, we explored the overall survival of genetically altered and unaltered groups via the “Survival” module in the cBioPortal online platform.

### 2.6. Analysis of Survival and Prognosis

Gene Expression Profiling and Interactive Analysis (GEPIA) is an interactive web tool used for the analysis of RNA-seq data from The Cancer Genome Atlas (TCGA) and Genotype-Tissue Expression projects (GTEx) [21]. The overall survival (OS) analysis and disease-free survival (DFS) analysis of the palmitoylation regulators were performed by GEPIA (http://gepia.cancer-pku.cn/detail.php, accessed on 28 October 2021). The “median” expression level divided patients into high and low groups. The log-rank *p*-values and hazard ratios (HRs) with 95% confidence intervals (CIs) were displayed in the plot. The *p* < 0.05 was considered statistically significant.

### 2.7. Relationship between ZDHHC7 Expression and Immunity

TIMER (Tumor Immune Estimation Resource) database (https://cistrome.shinyapps.io/timer/, accessed on 5 November 2021) is a web server utilized to analyze the correlation of gene expression with the abundance of immune infiltrates among diverse cancers [22]. We explored the correlation between the *ZDHHC7* expression and the immune infiltration levels of B cells, CD4(+) T cells, CD8(+) T cells, macrophages, dendritic cells and neutrophils among multiple types of cancers.

Tumor-infiltrating immune cells have crucial functions in regulating and/or promoting tumor progression [23]. So, we analyzed the correlation between *ZDHHC7* expression and 22 types of infiltrating immune cells in the TME of 39 cancer types via the “CIBERSORT” module in the Sangerbox (http://sangerbox.com/Tool, accessed on 12 November 2021). The Cell-type Identification by Estimating Relative Subsets of RNA Transcripts (CIBERSORT) [24] algorithm was applied to assess the relationship between *ZDHHC7* expression and 22 types of infiltrating immune cells based on the expression file. These immune cell subtypes include naïve B cells, memory B cells, plasma cells, CD8 T cells, CD4-naïve T cells, CD4 memory resting T cells, CD4 memory activated T cells, follicular helper T cells, regulatory T cells (Tregs), gamma delta T cells, resting NK cells, activated NK cells, monocytes, macrophages M0, macrophages M1, macrophages M2, resting dendritic cells activated dendritic cells, resting mast cells, activated mast cells, eosinophils and neutrophils.

Tumor mutational burden (TMB), microsatellite instability (MSI) and neoantigens are considered important biomarkers for helping develop immune checkpoint therapies [25,26,27]. TMB is defined as the total number of somatic gene-coding mutations, in which the detected variants are defined as deletion errors or gene insertions present in tumor tissue [28]. MSI is a molecular tumor phenotype that results from the loss of DNA mismatch repair activity [29]. We first obtained *ZDHHC7* expression and TMB, MSI, neoantigens data from Sangerbox. We calculated the Pearson correlation coefficient between *ZDHHC7* expression and TMB or MSI or neoantigens in the R software and the results were visualized with the “radar” (version 1.0.0) package.

### 2.8. Immune Checkpoint (ICP) Genes and ESTIMATE Score in Human Cancers

The TME is a heterogeneous population of cells in which infiltrating stromal and immune cells are foremost members of the TME [30,31,32]. The Estimation of STromal and Immune cells in MAlignant Tumours using Expression data (ESTIMATE) algorithm was used to calculate the *ZDHHC7* expression signature to evaluate the infiltration of stromal and immune cells in tumor samples [33]. The immune checkpoint genes have a major impact on the development of immune checkpoint therapies [34]. We explored the relationship between *ZDHHC7* expression and immune checkpoint (ICP) genes or ESTIMATE score across 33 cancer types via the SangerBox. These cancer types include adrenocortical carcinoma (ACC); bladder urothelial carcinoma (BLCA); breast invasive carcinoma (BRCA); cervical squamous cell carcinoma and endocervical adenocarcinoma (CESC); cholangiocarcinoma (CHOL); cervical squamous cell carcinoma (CESC); COAD; esophageal carcinoma (ESCA); GBM; head and neck squamous cell carcinoma (HNSC); KICH; KIRC; KIRP; acute myeloid leukemia (LAML); LGG; LIHC; lung adenocarcinoma (LUAD); lung squamous cell carcinoma (LUSC); mesothelioma (MESO); OV; pancreatic adenocarcinoma (PAAD); pheochromocytoma and paraganglioma (PCPG); prostate adenocarcinoma (PRAD); rectum adenocarcinoma (READ); sarcoma (SARC); skin cutaneous melanoma (SKCM); stomach adenocarcinoma (STAD); TGCT; thyroid carcinoma (THCA); thymoma (THYM); uterine corpus endometrioid carcinoma (UCEC); uterine carcinosarcoma (UCS); uveal melanoma (UVM). ICP genes were selected according to a previous study [35]. The data included three kinds of scores (ImmuneScore, StromalScore, and ESTIMATEScore) with *ZDHHC7* expression obtained from the Sangerbox. The results were visualized by the “ComplexHeatmap” (version 1.10.2) package in the R software [33].

### 2.9. Gene Set Enrichment Analysis

GSEA [36] was performed to screen the involved biological function of *ZDHHC7* in human development and cancer. We used the “h.all.v7.5.1.entrez.gmt” from the Molecular Signature Database (MSigDB) [37] as reference gene sets for GSEA. The Spearman correlation coefficients (SPCC) between the expression of *ZDHHC7* and other protein coding genes were calculated. All the genes were ranked by SPCC and analyzed by the “clusterProfiler” (version 3.0.4) package in the R program [38]. The *p*-adjust < 0.05 was considered statistically significant.

## 3. Results

### 3.1. Dynamic Process of Palmitoylation Regulators across Human Organ Development

**Figure 1:**
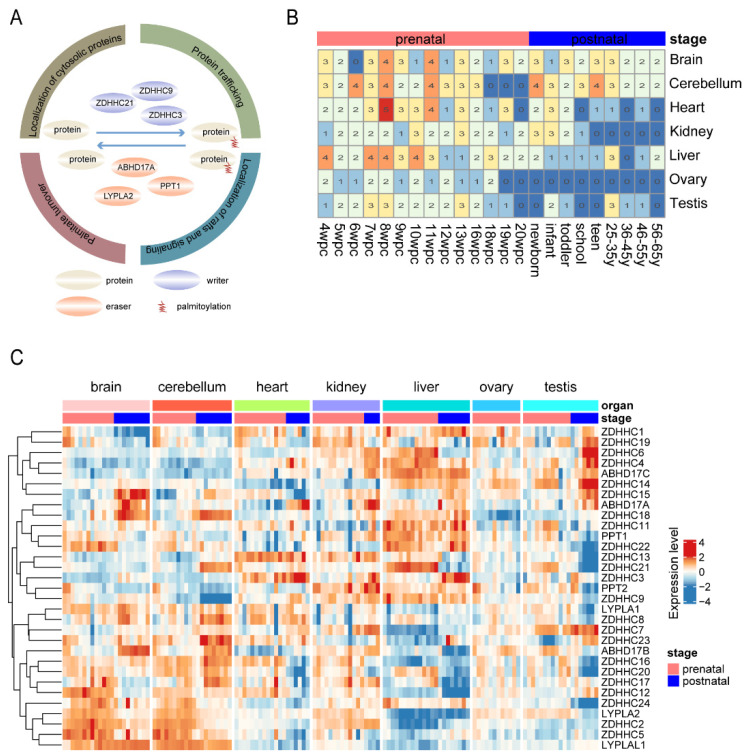
Dynamic expression of palmitoylation regulators during organ development. (**A**) Summary of regulation of palmitoylation modification. (**B**) Generalization of the number of samples at different developmental periods for seven organs. (**C**) Heatmap showing the expression levels of palmitoylation regulators in different organs. The prenatal and postnatal samples are shown in pink and blue colors.

Protein palmitoylation is a reversible post-translational modification that plays an essential role in biological processes, including human organ development and cancer progression (Figure 1A). To explore the dynamic expression of palmitoylation regulators across human organ development, we retrieved the data of 297 human samples involving seven organs and 24 time-points from public database. We divided all samples into prenatal groups and postnatal groups based on the developmental process of human organs (Figure 1B).

Based on the gene expression of palmitoylation regulators, the samples from different human organs were clustered. The expression levels of most palmitoylation regulators changed significantly in prenatal and postnatal samples. For example, we obtained that the expression of *ZDHHC15*, *ABHD17A*, and *ZDHHC18* in postnatal samples in seven organs is higher than that in prenatal samples (Figure 1C), while *ZDHHC20* and *ZDHHC17* exhibited decreased expression after birth (Figure 1C). Taken together, all these results imply that palmitoylation may have changed considerably during human organ development.

### 3.2. ZDHHC7/20/21 Present Significant Changes in Postnatal Samples

**Figure 2:**
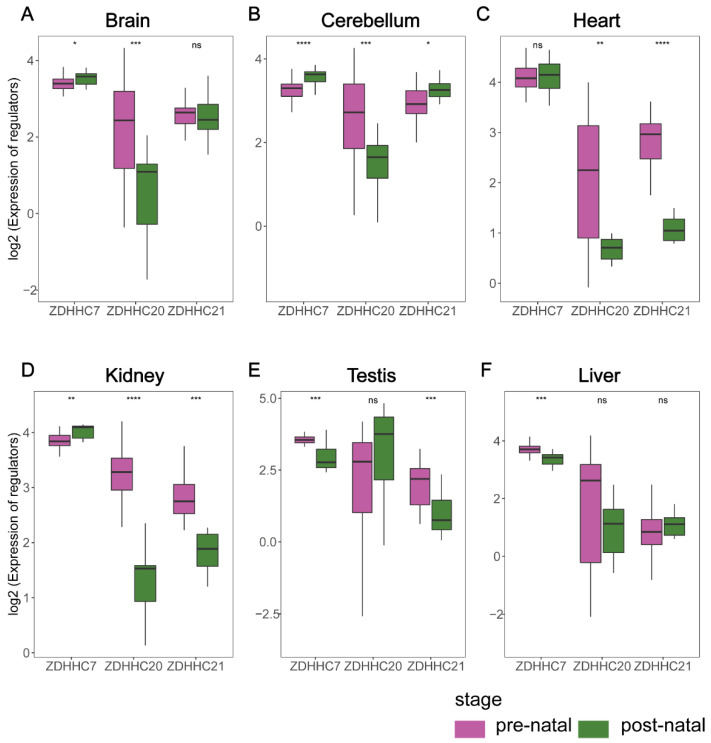
Boxplots exhibiting the expression of *ZDHHC7/20/21* in prenatal and postnatal samples of different organs. (**A**) Expression of *ZDHHC7/20/21* in the brain. (**B**) Expression of *ZDHHC7/20/21* in the cerebellum. (**C**) Expression of *ZDHHC7/20/21* in the heart. (**D**) Expression of *ZDHHC7/20/21* in the kidney. (**E**) Expression of *ZDHHC7/20/21* in testis. (**F**) Expression of *ZDHHC7/20/21* in the liver. * indicates *p* < 0.05. ** indicates *p* < 0.01. *** indicates *p* < 0.001. “ns” represents non-significant.

By analyzing the differential expression heatmap of palmitoylation regulators, we found that most palmitoylation regulators exhibited distinct changes in postnatal samples compared with prenatal samples. Specifically, the expression levels of *ZDHHC7/20/21* were significantly changed after birth (Figure 2). Then, a comprehensive analysis of the expression levels of *ZDHHC7/20/21* across six human organs was performed. In the brain, the expression level of *ZDHHC7* was higher in postnatal samples, while *ZDHHC20* was the opposite (Figure 2A). We found that the expression levels of *ZDHHC7* and *ZDHHC21* were significantly higher in postnatal samples in the cerebellum, while *ZDHHC20* showed a decreased expression after birth (Figure 2B). For heart, *ZDHHC20* and *ZDHHC21* showed a lower expression in postnatal samples (Figure 2C). Nonetheless, the expression levels of *ZDHHC20* and *ZDHHC21* in the kidney decreased after birth, while *ZDHHC7* expression increased in postnatal samples (Figure 2D). In the testis, *ZDHHC7* and *ZDHHC21* showed higher expression in prenatal samples (Figure 2E). For liver, the expression level of *ZDHHC7* decreased after birth (Figure 2F). Moreover, we examined the function of *ZDHHC7*, *ZDHHC20* and *ZDHHC21* in human organ development and cancer using PubMed in NCBI. Most studies in search results have explored their function in cancer, compared to few studies in organ development. Nicole et al. demonstrated that *ZDHHC7* deficiency changes the brain microstructure and connectivity in young people between 11 and 17 weeks of age, suggesting that palmitoylation plays an important role during the early stages of brain development [39]. Similarly, studies have shown that due to the *PPT1* expression pattern differing from the two other lysosomal enzymes implicated in NCL disease, *PPT1* has a distinctive role in brain development [8]. All these results suggest that changes in the expression levels of most palmitoylation regulators influence organ development.

### 3.3. Converse Changes in Expression Levels of ZDHHC7/20/21 in Cancer

**Figure 3:**
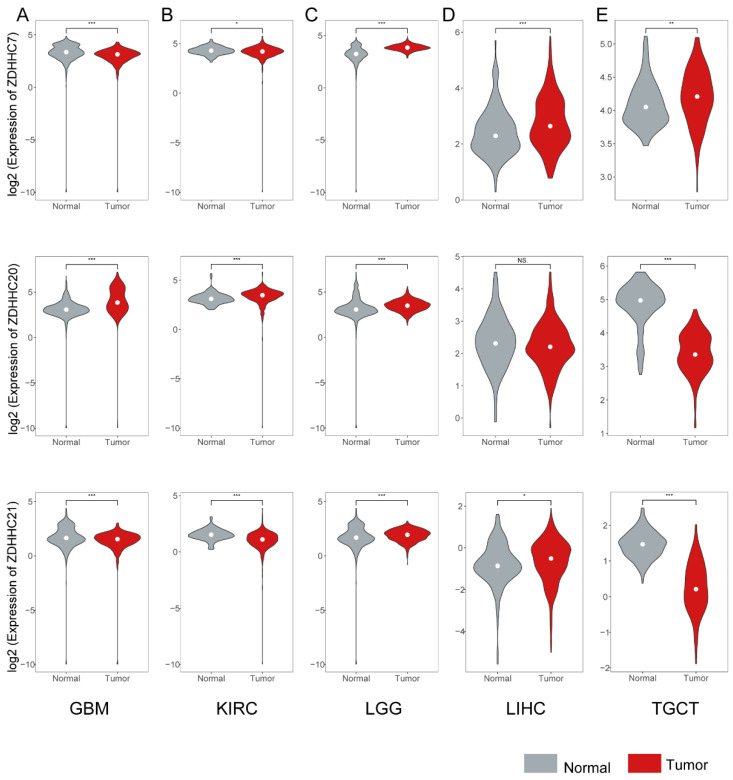
Violin plots exhibiting the expression of *ZDHHC7/20/21* in tumor and normal samples. (**A**) Expression of *ZDHHC7/20/21* in GBM. (**B**) Expression of *ZDHHC7/20/21* in KIRC. (**C**) Expression of *ZDHHC7/20/21* in LGG. (**D**) Expression of *ZDHHC7/20/21* in LIHC. (**E**) Expression of *ZDHHC7/20/21* in TGCT. * indicates *p* < 0.05. ** indicates *p* < 0.01. *** indicates *p* < 0.001. “NS.” represents no significance. A white dot represents the “median” value.

There has been an increasing number of researches linking cancer to the dysregulated protein palmitoylation [40,41,42,43]. Additionally, palmitoylation regulators exhibited abnormal expression in cancers. Several studies have revealed that Scribble without ZDHHC7-mediated palmitoylation is mislocalized, resulting in disruption of cell polarity and loss of its tumor suppressor activity in oncogenic pathways [44]. *ZDHHC21* was differentially expressed in COAD patients, which suggested its potential role in COAD initiation and progression [45]. Thus, we performed a systematic differential analysis between normal and tumor samples across eight cancer types. In comparison with organ development, the expression of *ZDHHC7/20/21* showed opposite expression patterns in eight cancer types (Figure 3, Supplementary Figure S1). For instance, the results indicated that *ZDHHC7* and *ZDHHC21* exhibited a lower expression in GBM, KIRC and OV patients, while *ZDHHC20* had a higher expression (Figure 3, Supplementary Figure S1). Besides, *ZDHHC20* and *ZDHHC21* exhibited a lower expression in TGCT, KICH and KIRP patients, while *ZDHHC7* only showed lower expression in TGCT patients (Figure 3, Supplementary Figure S1). These results indicated that expression of *ZDHHC7/20/21* showed opposite expression patterns in eight cancer types compared to in organ development, which implied that *ZDHHC7/20/21* might play essential roles in oncofetal reprogramming in cancer.

### 3.4. Prognostic Analysis of ZDHHC7/20/21 in Cancers

**Figure 4:**
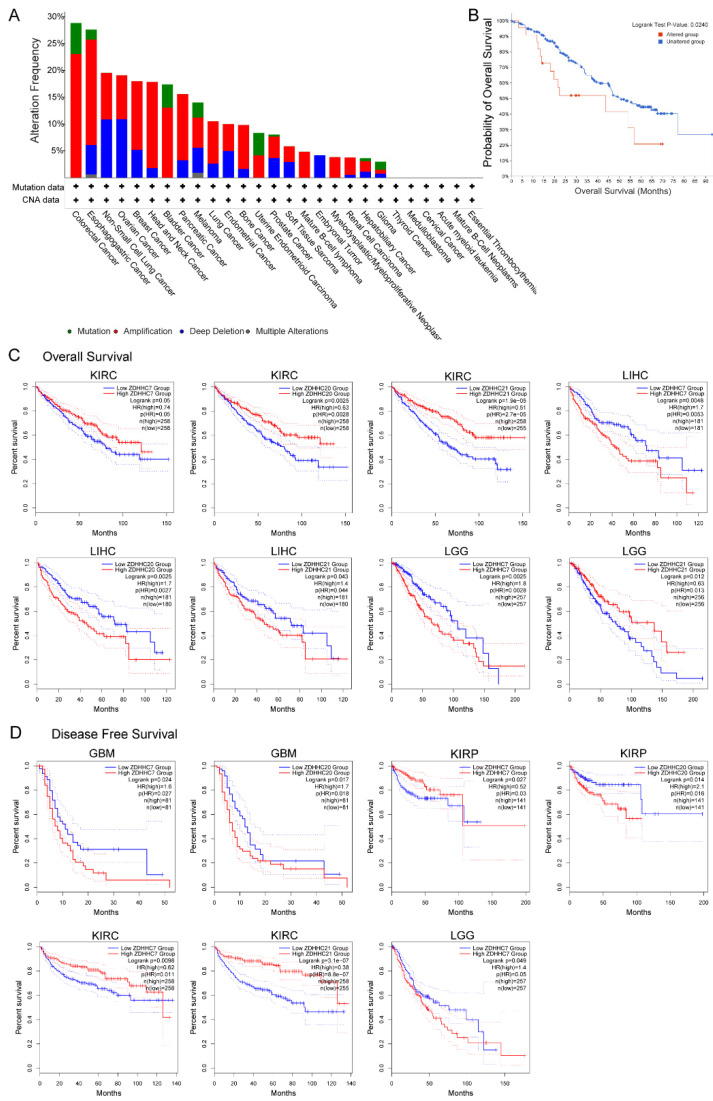
Genome alterations and Kaplan–Meier survival curve of *ZDHHC7/20/21* in human cancers. (**A**) Barplots showing the genetic alterations of *ZDHHC7/20/21* across cancer types. (**B**) Kaplan–Meier survival analysis of cancer patients in the *ZDHHC7/20/21* altered and unaltered groups. (**C**) The survival curve of *ZDHHC7* for OS in KIRC, LGG, and LIHC. (**D**) The survival curve of *ZDHHC7* for DFS in GBM, KIRP, KIRC, and LGG.

We first investigated the mutation frequency of *ZDHHC7/20/21* across various cancers via the cBioPortal database. The results indicated that *ZDHHC7/20/21* had a relatively high alteration frequency in colorectal cancer, esophagogastric cancer, and non-small cell lung cancer (Figure 4A). Next, we analyzed the association between the genetic alterations of *ZDHHC7/20/21* and OS. The results showed that the altered group had poorer survival than the unaltered group (Figure 4B, log-rank test *p* = 0.0240). Moreover, we further explored the relationships between the expression of *ZDHHC7/20/21* and the prognosis of cancer patients in multiple cancers using the GEPIA website (Figure 4C,D). Higher expression levels of *ZDHHC7* showed worse OS in LGG (*p* = 0.0025) and LIHC (*p* = 0.0048), worse DFS in GBM (*p* = 0.024) and LGG (*p* = 0.049), while showing a better OS in KIRC (*p* = 0.05) and DFS in KIRP (*p* = 0.027) and KIRC (*p* = 0.0098). Increased *ZDHHC20* expression was also linked with a poorer OS in LIHC (*p* = 0.0025), poorer DFS in GBM (*p* = 0.017) and KIRP (*p* = 0.027), while linked with a better OS in KIRC (*p* = 0.0025). Patients with higher *ZDHHC21* expression had better OS in KIRC (*p* = 1.9 × 10^−5^) and LGG (*p* = 0.012), better DFS in KIRC (*p* = 3.1 × 10^−7^), while they had worse OS in LIHC (*p* = 0.043).

To sum up, the above results showed that *ZDHHC7/20/21* expression is significantly related to the prognosis of patients, especially in those with KIRC, LGG and LIHC. In addition, in many other cancer types such as LAML, LUSC, and UVM (Supplementary Figure S2), high *ZDHHC7* expression meant worse prognosis, which implies that *ZDHHC7* was a potential cancer biomarker.

### 3.5. ZDHHC7 Expression Is Related to Tumor Mutational Burden (TMB), Microsatellite Instability (MSI), Neoantigen, and ESTIMATE Score

**Figure 5:**
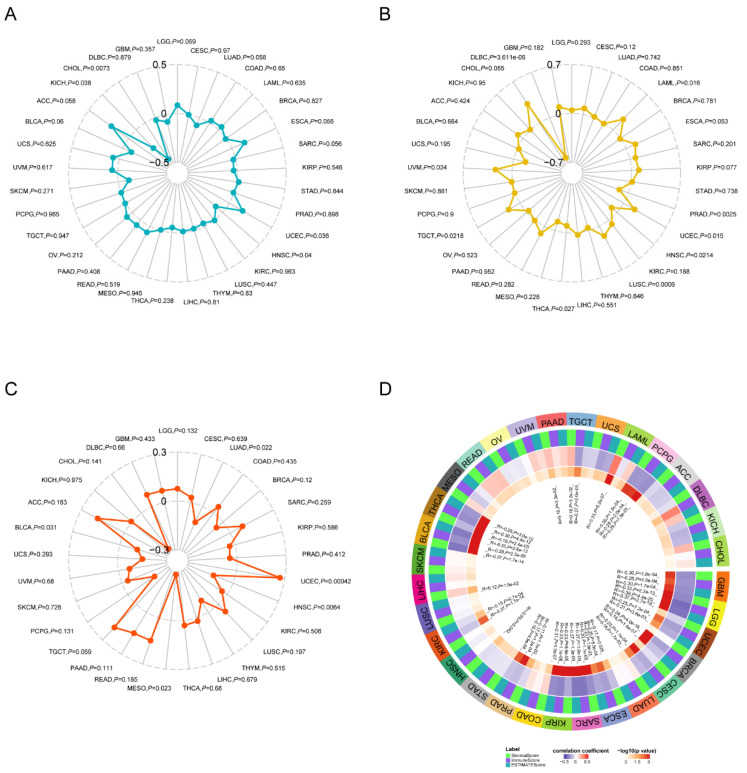
The relationship between *ZDHHC7* expression and tumor mutational burden (TMB) (**A**), microsatellite instability (MSI) (**B**), neoantigen (**C**) and ESTIMATE score (**D**) in human cancers. ACC, adrenocortical carcinoma; BLCA, bladder urothelial carcinoma; BRCA, breast invasive carcinoma; CESC, cervical squamous cell carcinoma and endocervical adenocarcinoma; CHOL, cholangiocarcinoma; CESC, cervical squamous cell carcinoma; COAD, colon adenocarcinoma; ESCA, esophageal carcinoma; GBM, glioblastoma multiforme; HNSC, head and neck squamous cell carcinoma; KICH, kidney chromophobe; KIRC, kidney renal clear cell carcinoma; KIRP, kidney renal papillary cell carcinoma; LAML, acute myeloid leukemia; LGG, brain lower grade glioma; LIHC, liver hepatocellular carcinoma; LUAD, lung adenocarcinoma; LUSC, lung squamous cell carcinoma; MESO, mesothelioma; OV, ovarian serous cystadenocarcinoma; PAAD, pancreatic adenocarcinoma; PCPG, pheochromocytoma and paraganglioma; PRAD, prostate adenocarcinoma; READ, rectum adenocarcinoma; SARC, sarcoma; SKCM, skin cutaneous melanoma; STAD, stomach adenocarcinoma; TGCT, testicular germ cell tumors; THCA, thyroid carcinoma; THYM, thymoma; UCEC, uterine corpus endometrioid carcinoma; UCS, uterine carcinosarcoma; UVM, uveal melanoma.

TMB, MSI and neoantigens are a part of tumor microenvironment and predictors of the response to immunotherapy [46,47]. Our previous analysis suggests that *ZDHHC7* may be a potential prognostic pan-cancer biomarker, so we evaluated the relationship between *ZDHHC7* expression and TMB, MSI and neoantigens. Here, we calculated the Pearson correlation coefficient between *ZDHHC7* expression and TMB, MSI and neoantigens across multiple cancers. The results represented that *ZDHHC7* expression was notably positive associations with TMB in UCEC, and negative relations in HNSC, KICH, and CHOL (Figure 5A, Supplementary Table S1). For MSI, the expression level of *ZDHHC7* was positively related to LAML, UCEC, LUSC, TGCT, and UVM, while it was negatively correlated with PRAD, HNSC, THCA, and DLBC (Figure 5B, Supplementary Table S1). Moreover, there was a significant positive correlation between *ZDHHC7* expression and neoantigens in UCEC, and negative relations in LUAD, MESD, HNSC, and BLCA (Figure 5C, Supplementary Table S1). In addition, we explored the relationships between *ZDHHC7* expression and three kinds of ESTIMATE score (Figure 5D, Supplementary Table S1). As shown in Figure 5D, *ZDHHC7* expression was positively correlated with three kinds of ESTIMATE score in LIHC, PAAD, TGCT, LAML, and DLBC and negatively correlated with three kinds of ESTIMATE score in GBM, LGG, UCEC, BRCA, CESC, ESCA, SARC, KIRP, PRAD, HNSC, KIRC, BLCA, THCA, and PCPG (Figure 5D, Supplementary Table S2). The above results imply that *ZDHHC7* may affect antitumor immunity by regulating the immune mechanisms in the TME.

### 3.6. Relationships between ZDHHC7 Expression and Immune Infiltrating Levels, Immune Checkpoint Genes in Cancers

**Figure 6:**
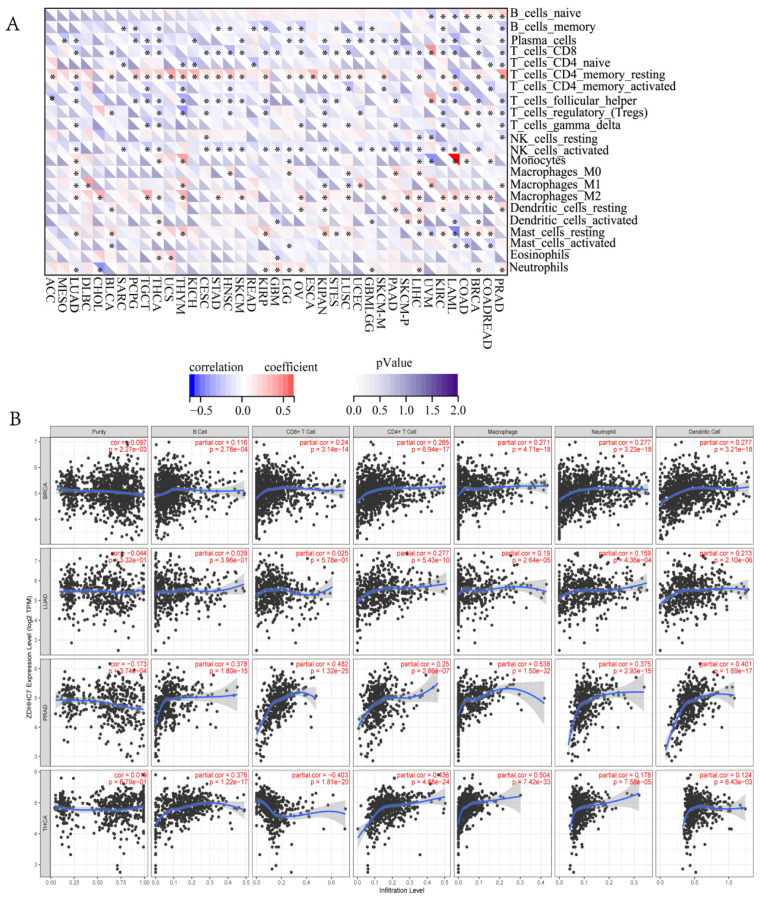
Correlation of *ZDHHC7* expression with immune infiltration levels across cancer types. (**A**) Correlation of *ZDHHC7* expression with the infiltration level of immune cells by the CIBERSORT algorithm. Immune cells positively correlating with *ZDHHC7* expression are labeled in red, and immune cells negatively correlating with *ZDHHC7* expression are labeled in purple. (**B**) Correlation of *ZDHHC7* expression with immune infiltration level in BRCA, LIHC, LUSC and PRAD.

**Figure 7:**
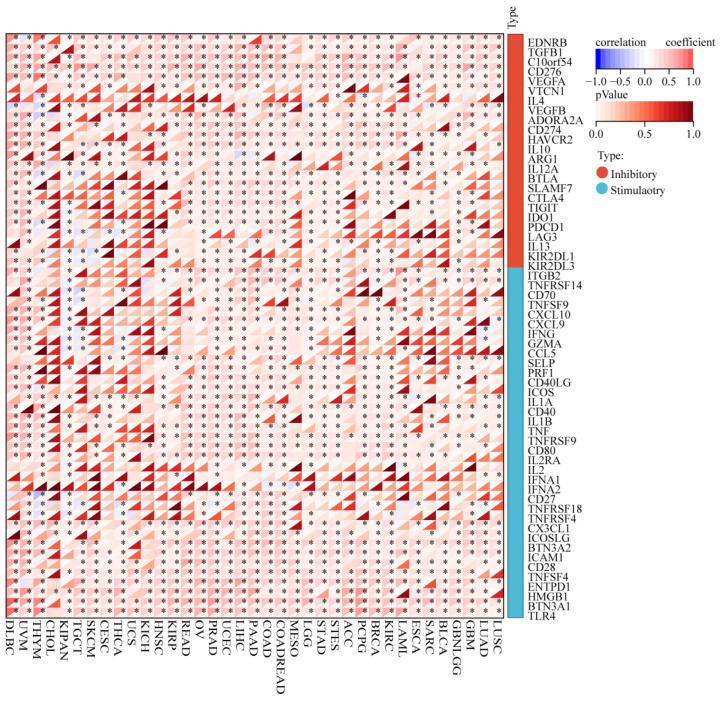
Correlation between *ZDHHC7* expression and 60 immune checkpoint gene expressions across cancer types. * indicates *p* < 0.05.

The TME is significantly associated with the prognosis of cancer patients, which is critical for recognizing immune modifiers of tumor progression and developing cancer immunotherapies [48,49]. Therefore, we explored the correlation between *ZDHHC7* expression and levels of immune cell infiltration across cancer types using the TIMER database [22]. The results revealed that *ZDHHC7* expression was significantly correlated with tumor purity in 11 cancer types. Moreover, we discovered that *ZDHHC7* expression had notable correlations with the infiltration levels of B cells in 16 cancer types, CD8+ T cells in 15 cancer types, CD4+ T cells in 17 cancer types, macrophages in 22 cancer types, neutrophils in 22 cancer types, and dendritic cells in 24 cancer types. The correlation between infiltration of 22 kinds of immune cell subtypes and *ZDHHC7* expression was analyzed using Sangerbox (Figure 6A). Next, we found that BRCA, LIHC, LUSC, and PRAD were the four cancer types most greatly correlated with *ZDHHC7* expression at the level of immune infiltration (Figure 6B). The results showed that CD4 memory resting T cells, macrophages M2, and follicular helper T cells were the three immune cell subtypes most highly correlated with *ZDHHC7* expression across multiple cancers. Considering that immunotherapies are an important therapy for reducing tumors, the relationship between the expression of *ZDHHC7* and the expression of 60 immune checkpoint genes was further analyzed. Our findings revealed that *ZDHHC7* expression was positively correlated with most immune checkpoint genes in multiple cancers (Figure 7).

### 3.7. Predicted Functions of ZDHHC7 in Cancers

**Figure 8:**
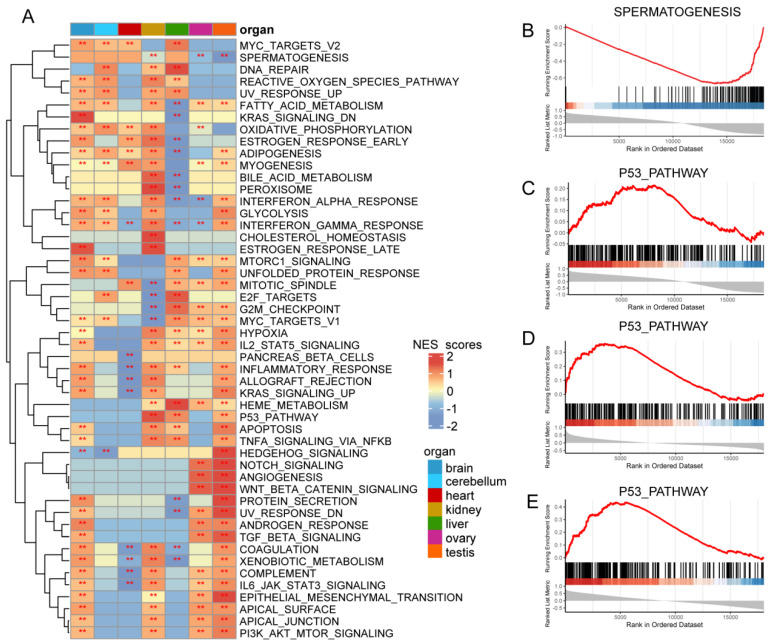
Gene set enrichment analysis of genes correlated with *ZDHHC7* in different organs. (**A**) Heatmap exhibiting the normalized enrichment scores of GSEA. Functional pathways with FDR < 0.05 ware shown in red stars. Each column represents one organ and each row represents one cancer-hallmark-related pathway. (**B**) GSEA plots exhibiting genes correlated with *ZDHHC7* in the spermatogenesis pathway in the testis. (**C**) GSEA plots exhibiting genes correlated with *ZDHHC7* in the p53 pathway in the liver. (**D**) GSEA plots exhibiting genes correlated with *ZDHHC7* in the p53 pathway in the testis. (**E**) GSEA plots exhibiting genes correlated with *ZDHHC7* in the p53 pathway in the kidney. ** represents *p*-adjust < 0.05 for GSEA.

In the previous analysis, we revealed the dynamic expression of *ZDHHC7* in human organ development and cancer. However, its function in development and cancer remains poorly explored. Thus, we predicted the biological function of *ZDHHC7* through GSEA. The results showed that genes correlated with *ZDHHC7* were significantly enriched in the MYC targets V1/V2, G2M checkpoint, mitotic spindle and E2F targets, which are involved in cancer cell proliferation, tumor growth, and tumor metastasis [50] (Figure 8A, Supplementary Table S3). In particular, genes correlated with *ZDHHC7* were significantly enriched in the spermatogenesis pathway in the testis (Figure 8B). Moreover, genes correlated with *ZDHHC7* were significantly enriched in the p53 pathway in the kidney, liver, and testis (Figure 8A).

Next, we investigated the p53 pathway in detail. We found that the expression of *ZDHHC7* was positively correlated with *TP53*, *SDC1*, and *RAP2B*, which were significantly enriched in the p53 pathway in the testis (Figure 8C, *p* < 0.001). For the liver, the expression of *ZDHHC7* was positively correlated with *CDKN2B*, *TGFB1* and *PIDD1*, which were enriched in the p53 pathway (Figure 8D, *p* < 0.001). Our findings indicated that *ZDHHC7* expression was positively correlated with *MKNK2*, *CD81*, and *CD82*, which were significantly enriched in the p53 pathway in the kidney (Figure 8E, *p* < 0.001). These results imply that *ZDHHC7* might regulate different genes in the same pathway and exert essential roles in cancer development.

## 4. Discussion

Increasing evidence has demonstrated that palmitoylation takes on an essential and widespread role in many cellular pathways and tumorigenesis [51,52]. Palmitoylation affects a variety of proteins and is a way of controlling their cellular trafficking and membrane localization. For example, the PDZ (*P*SD95-*D*lg-1-*Z*O) domain of *ZDHHC5* binds glutamate receptor-interacting protein (GRIP1b), promoting palmitoylation of the GRIP1 band and its subsequent trafficking to its dendritic localization [53]. As another example, *PPT1* is localized in lysosomes and late endosomes, separate from palmitoylase activity on the cytoplasmic matrix or plasma membrane [54]. As most studies focus on single cancer or single regulator, a comprehensive analysis of the dynamic expression of palmitoylation regulators in human organ development has yet to be fully elucidated. Therefore, it’s essential to investigate the roles of palmitoylation regulators on human organ development and multiple cancer types. Here, we revealed the dynamic expression of palmitoylation regulators during human organ development and cancers. We found that the expression levels of most palmitoylation regulators altered significantly after birth, with *ZDHHC7/20/21* showing opposite expression patterns in cancers. The above results suggest that *ZDHHC7/20/21* might play essential roles in oncofetal reprogramming in cancer.

Considering that the expression of palmitoylation regulators is widely dysregulated in multiple cancers, we explored the underlying mechanism for regulating their expression in cancer. For example, patients with overexpression of *PPT1* showed poorer survival in multiple cancers, suggesting the potential of *PPT1* inhibition strategies in cancer therapy [55]. Our study indicated that *ZDHHC20* exhibited a higher expression in GBM, KIRC, and LGG, consistent with a previous study in KIRC [56]. Studies have shown that the *ZDHHC20* expression was elevated in breast and lung cancer cell lines in which epidermal growth factor receptor (EGFR) signaling is present, which implied that *ZDHHC20* might play a key role in signal regulation during oncogenesis [57,58]. *ZDHHC21* expression was decreased in GBM, KIRC, and TGCT, while its expression was increased in LGG and LIHC. This is consistent with a previous study in KIRC [56]. These results indicated that *ZDHHC7/20/21* indeed show broad perturbations in cancer.

Since the expression levels of palmitoylation regulators were aberrant in many cancer types and some palmitoylation regulators were closely associated with tumor development, we next explored whether the abnormal expression of the palmitoylation regulators was related to patient survival. Pei et al. found that a higher level of *ZDHHC18* mRNA expression was associated with poorer survival in patients with ovarian cancer and malate dehydrogenase 2 (MDH2) palmitoylation, catalyzed by *ZDHHC18*, promoted the malignant progression of ovarian cancer [59]. BRCA patients with increased *ZDHHC22* expressions were correlated with better relapse-free survival and *ZDHHC22* suppressed proliferation by restraining the AKT/mTOR signaling pathway [60]. Liang et al. revealed that *ZDHHC19* was overexpressed in osteosarcoma cell lines and it might accelerate osteosarcoma proliferation and metastasis [61]. Patients with an elevated expression of *ZDHHC3* were associated with worse survival in BRCA, suggesting that *ZDHHC3* might be a cancer target [62]. Mohammed et al. found that inhibition of *LYPLA1* gene expression restrained cell proliferation, migration and invasion in vitro of non-small cell lung cancer (NSCLC) cell lines, suggesting that *LYPLA1* might be an effective therapeutic target for NSCLC cancer therapy [63]. The overexpression of *ZDHHC14* in gastric cancer (GC) promoted cancer progression and invasion of cancer cells and might be a promising therapeutic target for the management of GC [64]. Tian et al. suggested that *ZDHHC5* has oncogenic ability and contributes to the tumorigenesis of NSCLC, which could be a potential novel therapeutic target [65]. Chen et al. found that *ZDHHC17* was up-regulated in GBM and activation of JNK and p38 MAPK mediated by *ZDHHC17* promotes the malignant progression of GBM [66]. Similar to the previous study, KIRC patients with decreased *ZDHHC20/21* expressions were significantly associated with poor overall survival [56]. In addition, our results demonstrated that patients with a lower expression of *ZDHHC7/20/21* showed worse survival in LIHC. Liu et al. found that the group with lower *ZDHHC21* expression levels has higher disease-free survival possibilities than those with high *ZDHHC21* in OS [67]. Thus, these results implied that *ZDHHC7/20/21* play oncogenic roles in cancer.

The TME consists of tumor cells and the surrounding physical and cellular environment, which embodies infiltrating immune cells (IICs), cancer-associated fibroblastic cells (CAFs) and angiogenic vascular cells (AVCs) and influences cancer development and cancer progression [68,69,70]. Our findings demonstrated that *ZDHHC7* was highly associated with tumor-infiltrating immune cells. For example, *ZDHHC7* expression was significantly correlated with dendritic cells, macrophages, and neutrophils. As one of the most abundant stromal components in the TME, tumor-associated macrophages (TAMs) can promote tumor progression by facilitating tumor cell metastasis, invasion, suppressing antitumor immune responses, etc. [71]. Besides, MSI, TMB, and neoantigens are vital biomarkers of the TME [25,26,27]. Our study found that *ZDHHC7* negatively correlated with the immune, stromal and ESTIMATE scores of the TME in many cancer types. In addition, *ZDHHC7* expression was closely associated with TMB, MSI, neoantigens and immune checkpoint genes. Finally, GSEA analysis revealed that *ZDHHC7* might impact cancer progression by MYC targets V1/V2, G2M checkpoint, mitotic spindle and E2F targets pathway. Totally, these results suggest that *ZDHHC7* had close associations with the TME and provided novel insights into the treatment of cancers.

However, our study still has some limitations. Although the differential palmitoylation regulators’ expression was detected between normal and tumor samples, the prognostic significance of this finding can be further demonstrated. Second, in our current research, the transcriptome expression profile of palmitoylation regulators with clinical data was analyzed. More omics data, such as genomic, proteomic and epigenomic data, can be further explored. Third, the underlying mechanisms of palmitoylation regulators is also a potential research direction. Moreover, integration of these data will provide new insights into the roles of palmitoylation regulators in the development of cancer.

## Data Availability

Not applicable.

## Supplementary Materials

The following supporting information can be downloaded at: https://www.mdpi.com/article/10.3390/cimb44100306/s1.
