# Supplementary material for: Dynamic Expression of Palmitoylation Regulators across Human Organ Development and Cancers Based on Bioinformatics
Source: Curr Issues Mol Biol. 2022 Sep 27;44(10):4472–89. doi: 10.3390/cimb44100306 (PMC9600046; doi:10.3390/cimb44100306)
Supplement: Supplementary file 1 [file cimb-44-00306-s001.zip › Supplementary Figures.pdf]

Supplementary Figures

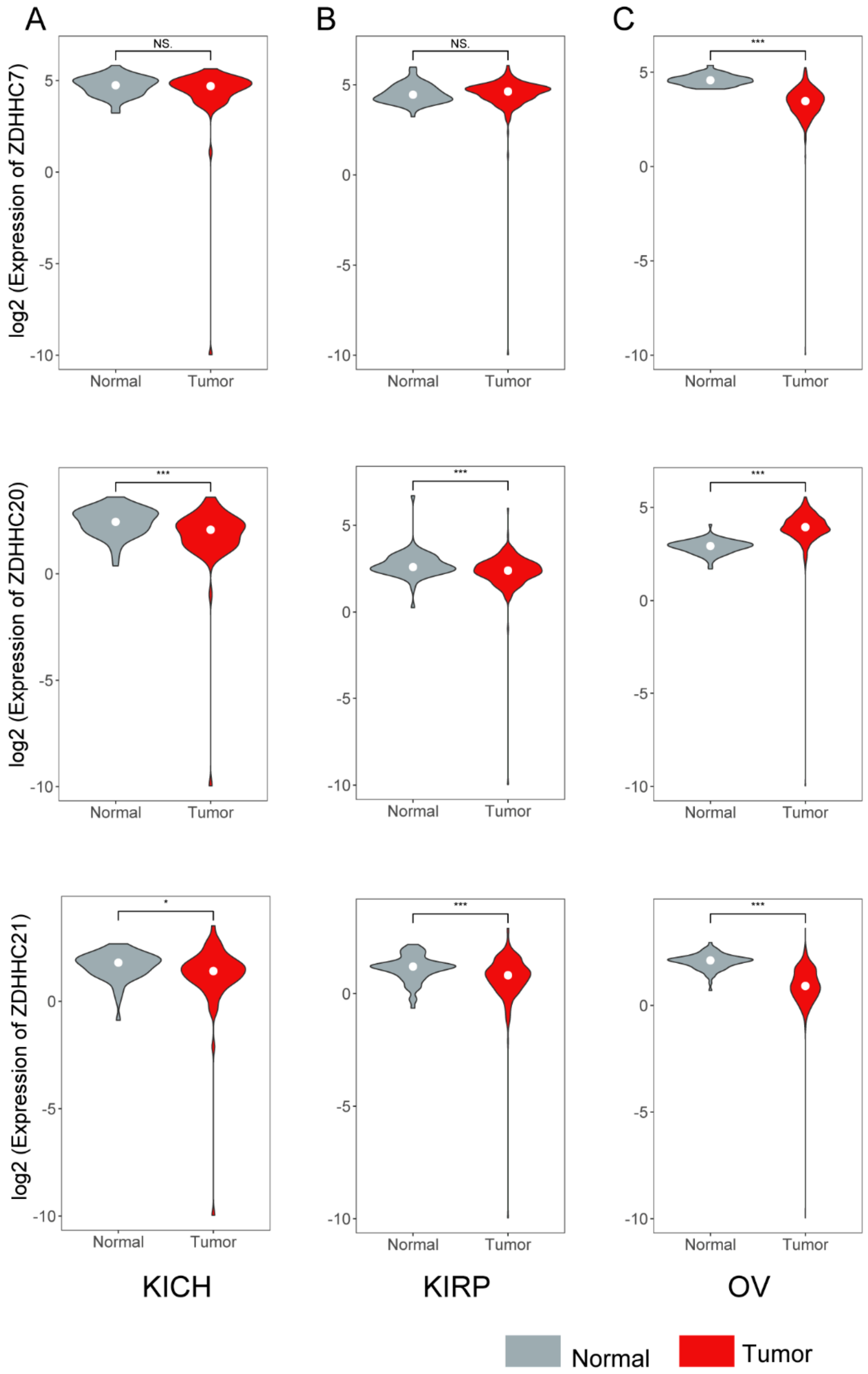

Figure S1. Expressions of ZDHHC7/20/21 across cancer types. * indicates $p < 0.05$. ** indicates $p < 0.01$. *** indicates $p < 0.001$. "NS." represents no significant. A white dot represents "median" value.

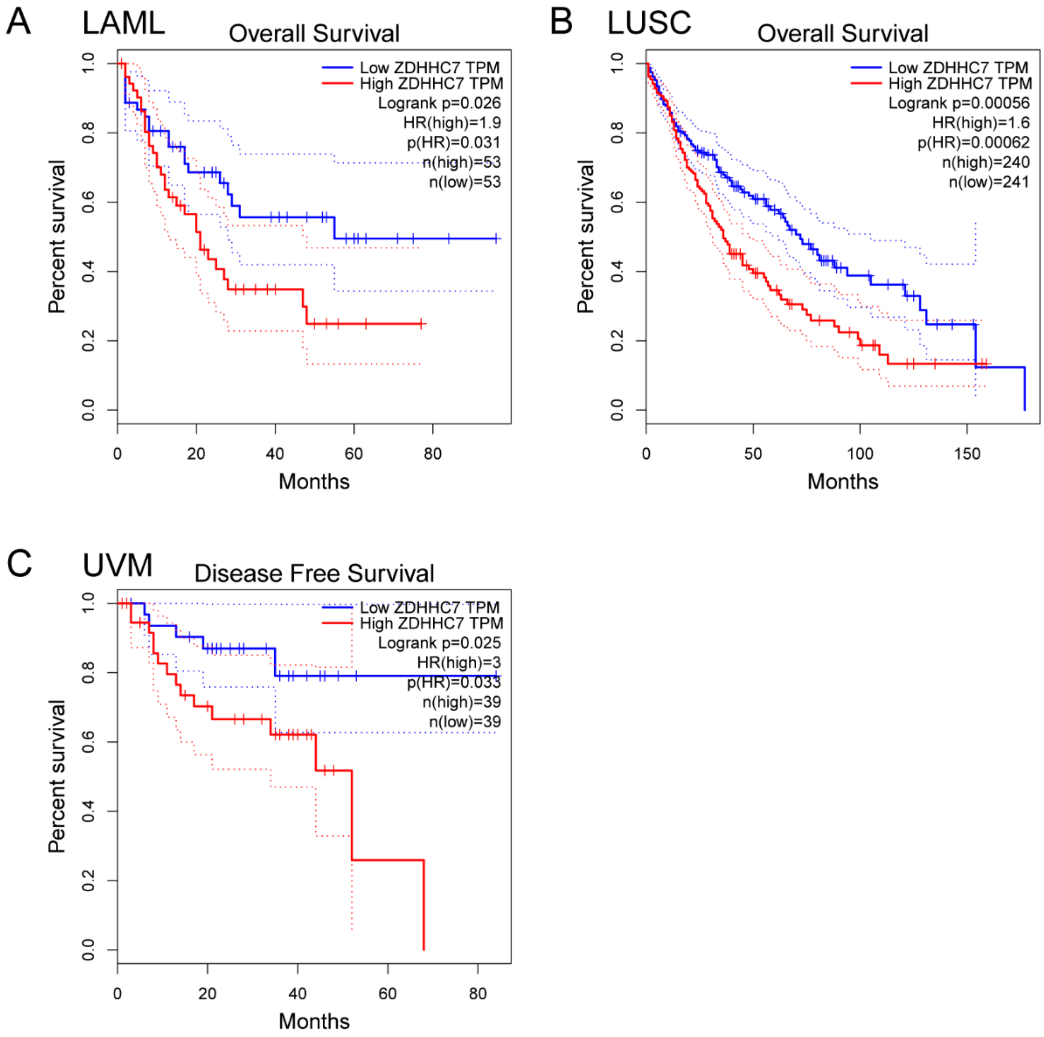

Supplementary Figure S2. Kaplan-Meier analysis of cancer patients in the ZDHHC7 high expression and low expression groups.
